# Right ventricular-pulmonary arterial coupling impairment and exercise capacity in obese adults

**DOI:** 10.3389/fcvm.2022.946155

**Published:** 2022-08-19

**Authors:** Na Zhou, Kevin Forton, Yoshiki Motoji, Corentin Scoubeau, Malgorzata Klass, Robert Naeije, Vitalie Faoro

**Affiliations:** ^1^Cardio-Pulmonary Exercise Laboratory, Faculty of Motor Science, Université Libre de Bruxelles, Brussels, Belgium; ^2^Department of Cardiology, Erasmus University Hospital, Brussels, Belgium; ^3^Laboratory of Applied Biology and Research Unit in Applied Neurophysiology, ULB Neuroscience Institute, Université Libre de Bruxelles, Brussels, Belgium; ^4^Laboratory for Biometry and Exercise Nutrition, Faculty of Motor Sciences, Université Libre de Bruxelles, Brussels, Belgium

**Keywords:** stress echocardiography, right ventricular-pulmonary arterial coupling, pulmonary circulation, pulmonary vascular resistance, pulmonary vascular reserve, VO_2_max = maximal oxygen uptake, obesity

## Abstract

**Background:**

Obesity-related exercise intolerance may be associated with pulmonary vascular and right ventricular dysfunction. This study tested the hypothesis that decreased pulmonary vascular reserve and right ventricular (RV)-pulmonary arterial (PA) uncoupling contributes to exercise limitation in subjects with obesity.

**Methods:**

Seventeen subjects with obesity were matched to normo-weighted healthy controls. All subjects underwent; exercise echocardiography, lung diffusing capacity (DL) for nitric oxide (NO) and carbon monoxide (CO) and an incremental cardiopulmonary exercise test. Cardiac output (Q), PA pressure (PAP) and tricuspid annular plane systolic excursion (TAPSE) were recorded at increasing exercise intensities. Pulmonary vascular reserve was assessed by multipoint mean PAP (mPAP)/Q relationships with more reserve defined by lesser increase in mPAP at increased Q, and RV-PA coupling was assessed by the TAPSE/systolic PAP (sPAP) ratio.

**Results:**

At rest, subjects with obesity displayed lower TAPSE/sPAP ratios (1.00 ± 0.26 vs. 1.19 ± 0.22 ml/mmHg, *P* < 0.05), DL_CO_ and pulmonary capillary blood volume (52 ± 11 vs. 64 ± 13 ml, *P* < 0.01) compared to controls. Exercise was associated with steeper mPAP-Q slopes, decreased TAPSE/sPAP and lower peak O_2_ uptake (VO_2_peak). The changes in TAPSE/sPAP at exercise were correlated to the body fat mass (*R* = 0.39, *P* = 0.01) and VO_2_peak (*R* = 0.44, *P* < 0.01).

**Conclusion:**

Obesity is associated with a decreased pulmonary vascular and RV-PA coupling reserve which may impair exercise capacity.

## Introduction

Obesity is associated with a risk of pulmonary hypertension (1). Echocardiographic studies have shown an increase in systolic pulmonary artery pressure (sPAP) in proportion to increased body mass index (BMI) (2, 3). The precise pathophysiology underlying obesity-induced pulmonary hypertension is complex and remains undefined (4).

Obesity is a cause of high output heart failure (5) and carries a specific predisposition to alterations in right ventricular (RV) structure and function (6, 7). Subjects with obesity display a higher RV mass and larger RV end-diastolic volumes as compared with normo-weighted controls (4, 7). Recent studies also showed extensive subclinical RV dysfunction in uncomplicated obese adults, which was independently associated with BMI and central fat mass distribution (6, 8).

An invasive cardiopulmonary exercise test (CPET) study recently reported on RV-pulmonary arterial (PA) uncoupling measured by gold standard ratio of end-systolic to arterial elastances (Ees/Ea) as a cause of obesity-related exercise intolerance (9). However, quantification of cardiorespiratory fitness in obese subjects is difficult because of variety of prediction equations and body mass corrections (10). How changes in pulmonary vascular function correlate to RV-PA uncoupling in mild to moderate obesity at rest and at exercise is not known.

A higher pulmonary vascular reserve defined as a lower pulmonary arterial pressure (PAP) at high cardiac output (Q) with a greater pulmonary capillary bed and diffusing capacity, has been shown to allow for superior aerobic capacity (VO_2_peak) in healthy subjects (11–13). The effects of obesity on the pulmonary vascular reserve and how it modulates aerobic exercise capacity is not exactly known.

We therefore undertook the present prospective study, which aimed to correlate exercise stress echocardiography indices of pulmonary vascular function and RV-PA coupling to body composition and CPET assessment of VO_2_peak in obese subjects as compared to lean counterparts. Pulmonary vascular function was assessed by multipoint mean PAP (mPAP)/cardiac output (Q) relationship to better discriminate flow-dependent changes in PAP or pulmonary vascular resistance (PVR) (14). The percentage of increase in the diameter of the resistive vessels of the pulmonary circulation per mmHg of increase of the transmural pressure during exercise was assessed by the calculation of a pulmonary vascular distensibility coefficient, α (15). Pulmonary capillary blood volume (Vc) was calculated from lung diffusing capacity (DL) for nitric oxide (NO) and carbon monoxide (CO) measurements (16). RV function adaptation to afterload was assessed by the ratio of tricuspid annular plane systolic excursion (TAPSE) to sPAP (17). Aerobic exercise capacity and gas exchange at exercise were assessed by CPET (18).

We hypothesized that this combination of non-invasive measurements might disclose decreased pulmonary vascular reserve and RV-PA uncoupling accounting for a decreased aerobic exercise capacity in subjects with obesity.

## Methods

### Study population

The study included 17 subjects with obesity, as defined by a BMI ≥ 30 kg/m^2^, and 17 healthy normo-weighted controls matched for sex, age, height and race. The 17 obese subjects had been pre-selected from a group of 37 for optimal echogenicity at rest and at exercise. All participants were non-smokers with a normal clinical examination. Exclusion criteria were conditions that contraindicated the performance of physical tests, systemic diseases (i.e., cancer, cardio-vascular or lung disease or skeletal conditions), and diseases that could alter balance and functional activity. However, 10 subjects with obesity declared to have hypertension, 9 asthma symptoms, 8 dyslipidemia, 6 sleep-apnea and 4 latent diabetes. The control subjects used no drugs and had normal clinical examination, blood pressure, electrocardiogram, and normal blood levels of glucose and lipids. All the subjects gave an informed written consent to the study which was approved by the local Ethical Committee of the University Hospital (Erasme EC Reference number: P2016/448; EC approval date: 2016-11-21). The general and anthropometric characteristics of the two study groups are summarized in Table 1.

**Table 1:** Baseline characteristics of subjects with obesity and controls.

	**Subjects with obesity**	**Controls**
	**(*n* = 17)**	**(*n* = 17)**
Men, %	29%	29%
Age, year	44 ± 11	46 ± 12
Height, cm	169 ± 9	169 ± 9
Body weight, kg	111 ± 17	64 ± 11***
BMI, kg/m^2^	38 ± 4.2	22 ± 2.1***
BSA, m^2^	2.2 ± 0.2	1.7 ± 0.2***
FM, % body weight	49 ± 5	30 ± 6***
VAT, g	1988 ± 1128	367 ± 429***
**Global physical activity questionnaire**		
Sedentary time (min/day)	567 ± 165	305 ± 271**
Global physical activity time (min/week)	357 ± 353	893 ± 978**
Vigorous activity time (min/week)	0 ± 0	177 ± 387***
Moderate activity time (min/week)	357 ± 353	717 ± 706*

BMI, body mass index; BSA, body surface area; FM, fat mass; VAT, visceral adipose tissue. ^*^P < 0.05, ^**^P < 0.01, ^***^P < 0.001; subjects with obesity vs. controls.

### Study design

Each subject underwent a sequence of clinical examination, body composition assessment, lung diffusing capacity measurements, exercise stress echocardiography, CPET and filled out the Global Physical Activity Questionnaire (GPAQ).

### Clinical assessment

Clinical assessment included a medical history, clinical examination with measurements of resting blood pressure (BP) (sphygmomanometry), ECG, pulsed oximetry (SpO_2_) (Nelcor Puritan Bennett Inc, Pleasanton, CA), blood sampling to measure hemoglobin for DL_CO_ correction and the GPAQ to assess self-estimated daily physical activity. The GPAQ allows for the estimation of the total weekly time of moderate- and vigorous- intensity physical activities (MVPA) in a typical week among these three domains: activity at work, active travel and recreational activities (19).

### Body composition assessment

All the measurements were performed in the morning after an overnight fast. Height was measured to the nearest 0.5 cm with a wall-mounted stadiometer. Weight was measured to the nearest 0.1 kg on a standing weighting scale (BC-418, TANITA, Japan) wearing no shoes and light clothing. BMI was expressed in kg/m^2^ where kg is the person's weight in kilograms and m^2^ is the height in meters squared. Total and regional fat mass (FM), lean mas (LM) and visceral adipose tissue (VAT) were acquired using dual energy X-ray absorptiometry (DEXA) and analyzed using enCORE (version 1.5) and CoreScan softwares (GE Healthcare, Madison, WI, USA).

### Lung diffusing capacity

DL_CO_ and DL_NO_ were measured using single breath methods in the sitting position with corrections for hemoglobin levels and inspired partial pressure of oxygen using an automated device for calibrations, mixing of gases and online calculations (Hyp'Air compact, Medisoft, Dinant, Belgium) as reported previously (13) and following updated international recommendations (20, 21).

### Exercise stress echocardiography

Resting and exercise echocardiography was performed with a commercially available portable system (CX50 CompactXtreme Ultrasound System; Philips, Amsterdam, The Netherlands) on a semi-recumbent cycle ergometer (model 900 EL; Ergoline) left tilted by 20° as previously reported (13) following international recommendations (22) and updated multi-centric experience (23). The workload was increased by 10–15 W every 2 min until exhaustion, and echocardiographic measurements were recorded during the last minute of each workload-step. The study focused on measurements of cardiac output (Q), calculated as stroke volume (SV) times heart rate (HR), SV estimated from resting left ventricular outflow tract cross-sectional area and the pulsed Doppler velocity-time integral, sPAP estimated from a calculated trans-tricuspid pressure gradient, left atrial pressure (LAP) estimated from the transmitral flow (E) to mitral annulus tissue Doppler e' velocities with the following equation: LAP = 1.9 + 1.24 E/e' (24), and TAPSE measure by M mode. Mean pulmonary artery pressure (mPAP) was calculated as 0.61 × (4 × TRV^2^ + 5 mmHg) + 2 mmHg. Total pulmonary vascular resistance (TPR) was calculated as mPAP/Q. The ΔTAPSE/sPAP calculated as the difference between peak exercise and rest values of the TAPSE/sPAP ratio was used to assess RV-PA coupling reserve. Pulmonary vascular reserve was estimated by the slope of multipoint mPAP/Q plots (25), a lower slope meaning more reserve. Pooled mPAP-Q relationships of each study group are illustrated in **Figure 3** after adjustment for individual variability as reported by Poon (26). A distensibility coefficient α, % diameter change per mmHg pressure was also calculated using the equation: mPAP = [(1 + αLAP)^5^ + 5αTPR.Q]^1/5^- 1 / α with measurements of mPAP, LAP, TPR and Q of each subject at rest (α-rest), at common maximal exercise (α-common peak) and including all mPAP/Q points (α-total), as reported previously (13–15). Inter-group comparison was performed at maximal exercise but also at common maximum exercise level, captured when the subjects with obesity and controls reached the greatest identical indexed Q (QI), to correct for physical fitness disparities.

### Cardio-pulmonary exercise test

Aerobic capacity was assessed using a classical incremental CPET on an electrically braked cyclo-ergometer (Ergoselect II 1200; Ergoline, Bitz, Germany) as previously reported (13) and following standard recommendations (27). VO_2_, CO_2_ output (VCO_2_) and ventilation (V_E_) were collected breath by breath through a facial mask and analyzed every 8 s using a metabolic system (Exp'Air^®^, Medisoft, Dinant, Belgium) calibrated with room air and standardized gas. The initial power started at 30 W for warm up with increments of 15–30 W/min, estimated from previous CPET performance and for an optimal test duration between 10 to 12 min until volitional exhaustion. Effort was considered maximal when two of the following criteria were met: VO_2_ increase >100 ml/min while workload further increases, respiratory exchange ratio (RER) > 1.10, achievement of age predicted maximal HR, incapacity to maintain the pedal rate ≥ 50 rpm. VO_2_peak was expressed in absolute value and relative to body weight. VO_2_peak was also predicted using the equation proposed by Wasserman et al. (27). The first ventilatory threshold (VT), used as a surrogate of aerobic exercise capacity, was determined by the V-slope method by two blinded independent experienced exercise physiologists. Ventilatory efficiency was assessed using the VE/VCO_2_ at VT.

### Statistical analysis

Data are expressed as mean ± standard deviation. *P* < 0.05 were considered significant. Normal distribution of the data was tested using the Shapiro-Wilk test. Normally distributed data were compared using unpaired *t*-test for the comparison of subjects with obesity vs. control subjects. A Wilcoxon rank-sum test was used for non-parametric inter-group comparison. Pearson correlation coefficient (r) was calculated to quantify the relation between two parameters.

Stress echocardiographic measurements in obese subjects and controls were compared at rest, at maximum exercise and at the maximum common QI. The mean angular coefficient of the individual linear mPAP/QI slopes of each study group were compared to investigate inter-group PVR differences.

Intra- and inter-observer variabilities for echocardiographic measurements were evaluated in 10 randomly selected subjects by the main observer and by a second independent observer at rest and at the maximum workload. The intra- and inter- observer variabilities exceeded 80% reliability.

Data analyses were conducted using GraphPad Prism 8 (GraphPad Software, California).

## Results

### Clinical assessment

Baseline clinical characteristics are described in Table 1. Both population groups were predominantly female (25% men), with a mean age of 44 to 47 years. The obese subjects had higher body weight, BMI, BSA, FM and VAT.

Daily sedentary time was longer in subjects with obesity and weekly global physical activity time, including vigorous activity time and moderate activity time, were lower.

### Exercise stress echocardiography

The results of pulmonary hemodynamic and RV function measurements are shown in Table 2.

**Table 2:** Hemodynamic at rest and at common maximal exercise level.

	**Subjects with obesity**	**Controls**
	**(*n* = 17)**	**(*n* = 17)**
**Rest**		
MAP, mmHg	98 ± 11	83 ± 8***
HR, bpm	72 ± 11	65 ± 8*
E/e' ratio	7.4 ± 1.0	6.5 ± 1.5*
Q, l/min	5.6 ± 1.7	4.5 ± 0.9*
QI, l/min/m^2^	2.5 ± 0.7	2.7 ± 0.6
mPAP, mmHg	16 ± 2	15 ± 2
LAP, mmHg	11.3 ± 1.5	9.9 ± 1.8
TPRI, Wood units.m^2^	6.8 ± 2.7	5.8 ± 1.6
TAPSE, mm	22 ± 2	24 ± 3*
TAPSE/sPAP, mm/mmHg	1.00 ± 0.26	1.15 ± 0.22*
α-rest, %/mmHg	2.7 ± 2.4	2.5 ± 1.5
**Exercise at normalized maximal common QI**		
LAP, mmHg	12.3 ± 1.3	10.2 ± 2.0
E/e' ratio	7.5 ± 1.1	7.2 ± 1.5
QI, l/min/m^2^	5.1 ± 1.2	5.1 ± 1.9
mPAP, mmHg	26 ± 5	22 ± 5*
TPRI, Wood units.m^2^	5.1 ± 1.1	4.3 ± 0.7**
TAPSE, mm	30 ± 3	32 ± 3*
TAPSE/sPAP, mm/mmHg	0.82 ± 0.19	1.01 ± 0.23**
α-common max, %/mmHg	1.3 ± 0.7	1.2 ± 0.5
α-total, %/mmHg	1.5 ± 0.5	1.4 ± 0.6

BSA, body surface area; MAP, mean arterial pressure; HR, heart rate; Q, cardiac output; E/e', the ratio of early diastolic mitral inflow to mitral annular tissue velocities; QI, cardiac index; mPAP, mean pulmonary artery pressure; TPRI, total pulmonary vascular resistance index; TAPSE, tricuspid annular plane systolic excursion; sPAP, systolic pulmonary artery pressure; ^*^P < 0.05, ^**^P < 0.01, ^***^P < 0.001: subjects with obesity vs. controls.

Compared with the control group, subjects with obesity at rest had higher mean systemic arterial pressure (MAP), heart rate (HR), Q and LAP and lower TAPSE or TAPSE/sPAP, while mPAP, QI and indexed TPR (TPRI) were not different.

**Figure 1:**
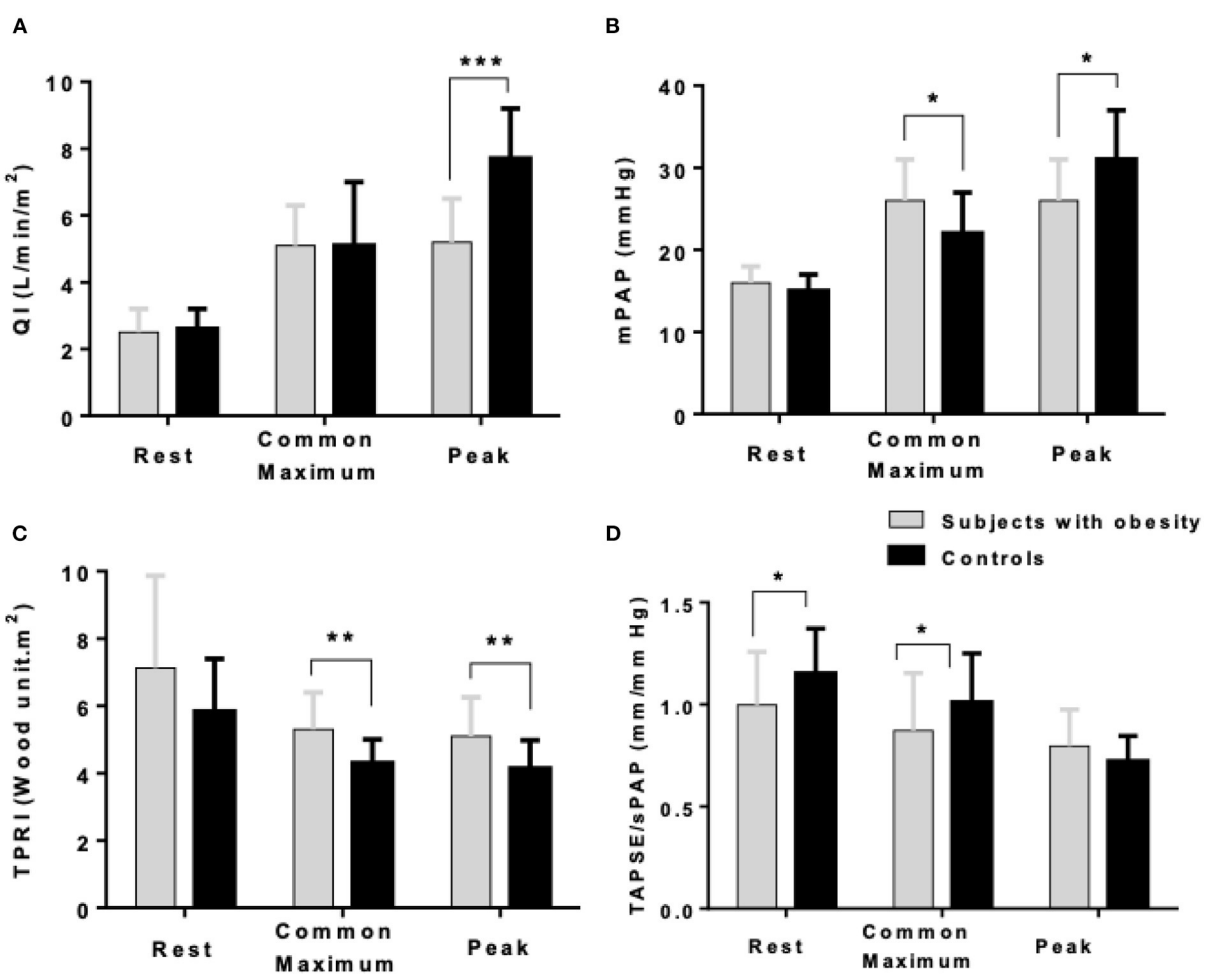
Cardiac index (QI) **(A)**, mean pulmonary artery pressure (mPAP) **(B)**, indexed total pulmonary vascular resistance (TPRI) **(C)**, and the ratio of the tricuspid annular plane systolic excursion by the systolic pulmonary artery pressure (TAPSE/sPAP) **(D)** at rest, common maximum exercise level and peak exercise in subjects with obesity and healthy controls. *P < 0.05, **P < 0.01, ***P < 0.001: subjects with obesity *vs*. controls.

**Figure 2:**
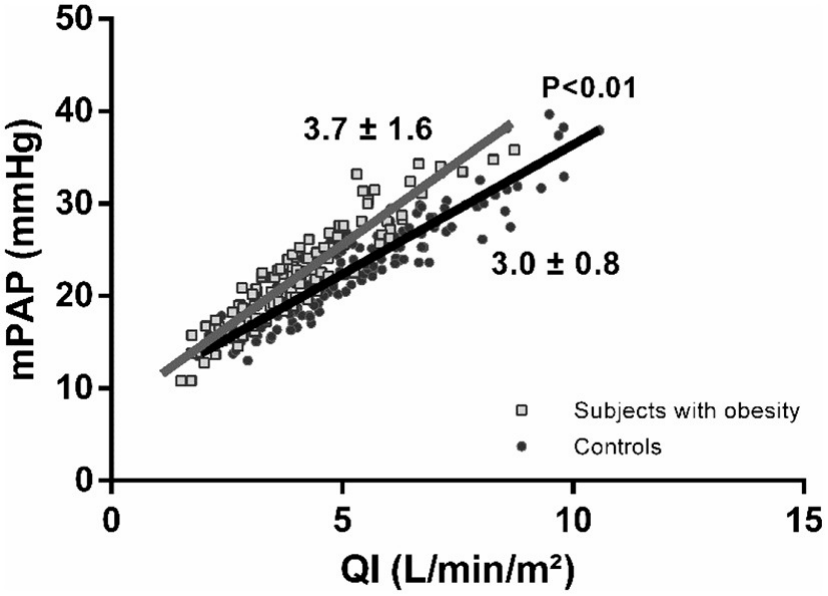
Poon-adjusted mean pulmonary artery pressure (mPAP)-cardiac index (QI) relationships in subjects with obesity (gray squares) vs. controls (black circles). Subjects with obesity present increased mPAP-QI slopes.

As shown in Figure 1, at maximum exercise, obese subjects had lower QI and mPAP but higher TPRI and TAPSE/sPAP. At common maximum exercise levels, QI and LAP were not different, but TAPSE and TAPSE/sPAP were lower and TPRI or mPAP higher in subjects with obesity. Resistive vessel distensibility α-total, calculated over the entire range of rest to exercise measurements, was not different. However, the slope of mPAP/QI was higher in the obese subjects (Figure 2).

**Figure 3:**
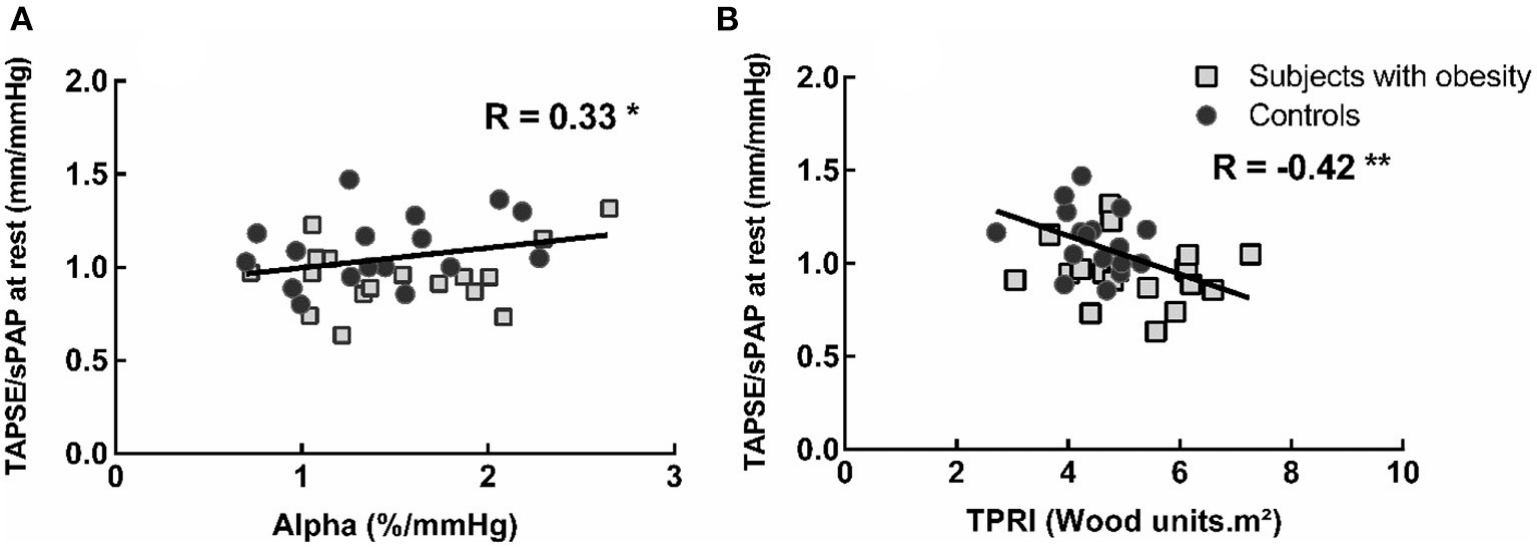
Correlation between the TAPSE/sPAP ratio at rest and the pulmonary vascular distensibility coefficient alpha-total during exercise **(A)** and exercise indexed total pulmonary vascular resistance (TPRi) at normalized maximal common QI **(B)**. Subjects with lower pulmonary vascular distensibility and higher TPRi present lower TAPSE/sPAP ratios. *P < 0.05 and **P < 0.01.

**Figure 4:**
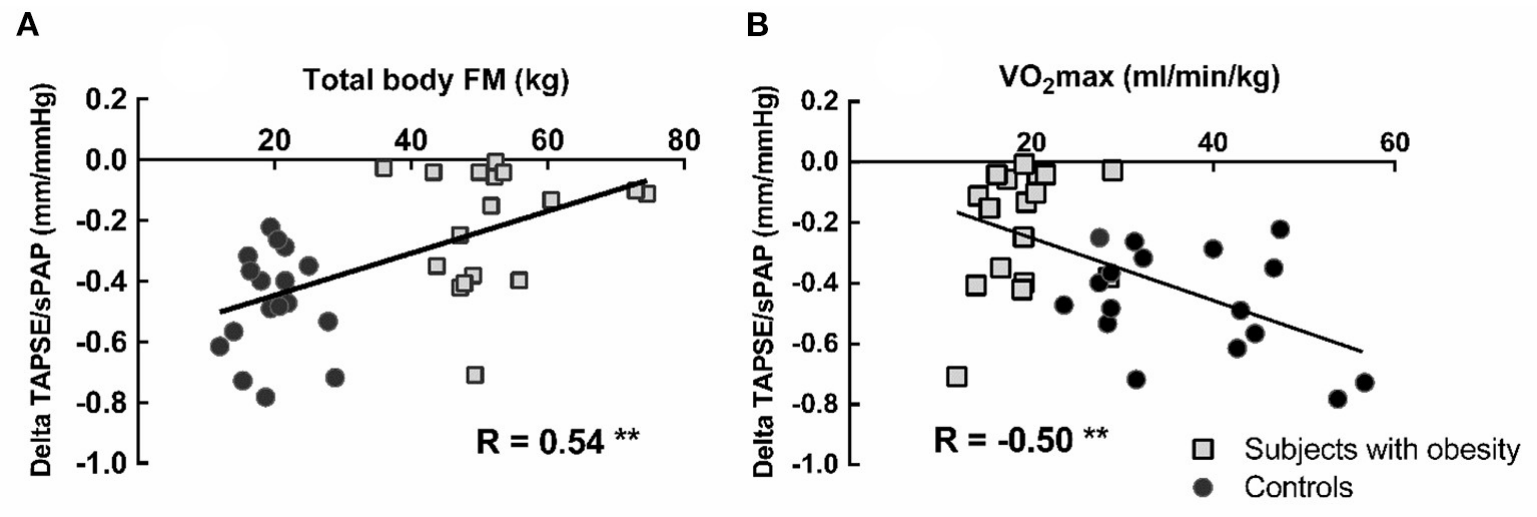
Correlation between total body fat mass (FM) and peak exercise induced changes in TAPSE/sPAP **(A)**. Correlation between peak oxygen uptake (VO_2_peak) and the change of the TAPSE/sPAP ratio from rest to peak exercise **(B)**. Square markers represent patients with obesity, and circle markers represent the healthy control subjects. Lower VO_2_peak was associated with shallower changes in TAPSE/sPAP ratios. **P < 0.01.

In the obese subjects and controls, resting TAPSE/sPAP was negatively correlated with TPRI at normalized maximal common QI and positively correlated with α-total (Figure 3). The changes in TAPSE/sPAP induced during exercise were correlated to the android fat mass, *R* = 0.49, *P* < 0.01 (not shown), the total body fat mass and the VO_2_peak (Figure 4).

### Lung diffusing capacity

Lung diffusion capacity measurements at rest are shown in Table 3. As compared with normo-weighted control group, subjects with obesity had a lower alveolar volume (V_A_), DL_CO_ and Vc corrected for the Hb level.

**Table 3:** Lung diffusion capacity at rest.

	**Subjects with obesity**	**Controls**
	**(*n* = 17)**	**(*n* = 17)**
V_A_, l	5.6 ± 1.2	6.4 ± 1.2**
DL_CO_ cor, ml/min/mmHg	24 ± 5	28 ± 5**
DL_NO_, ml/min/mmHg DL_NO_/DL_CO_	130 ± 26 5.5 ± 1.0	142 ± 27 5.0 ± 0.7
K_CO_, ml/min/mmHg/l	4.5 ± 0.9	4.4 ± 0.9
K_NO_, ml/min/mmHg/l	23.5 ± 2.8	22.6 ± 4.2
Dm, ml/min/mmHg	163 ± 44	171 ± 57
Vc cor, ml	52 ± 11	64 ± 13**

V_A_, alveolar volume; DL_CO_ cor, lung diffusion capacity for carbon monoxide corrected for Hb; DL_NO_, lung diffusion capacity for nitric oxide; K_CO_, DL_CO_ corrected for Hb and V_A_; K_NO_, DL_NO_ corrected for VA; Dm, membrane component of diffusion capacity; Vc cor, pulmonary capillary blood volume corrected for Hb. ^**^P < 0.01: subjects with obesity vs. controls.

### Cardio-pulmonary exercise testing

CPET results are displayed in Table 4. Subjects with obesity had a lower VO_2_peak in % predicted as well as relative to body weight, and increased VE/VCO_2_ but similar absolute VO_2_peak, and lower maximal workload, HR and V_E_ as compared to controls.

**Table 4:** Cardio-Pulmonary Exercise Tests in subjects with obesity and controls.

**Maximal exercise**	**Subjects with obesity**	**Controls**
	**(*n* = 17)**	**(*n* = 17)**
Workload, watt	134 ± 48	180 ± 50**
VO_2_peak, ml/min/kg	18 ± 5	38 ± 9***
VO_2_peak, l/min	2.0 ± 0.5	2.4 ± 0.7
VO_2_peak, % predicted	88 ± 23	124 ± 18
HR, bpm	156 ± 20	173 ± 15**
V_E_, l/min	76 ± 21	90 ± 24**
O_2_Pulse, ml/bpm	14.9 ± 3.0	16.4 ± 5.5
SpO_2_, %	97 ± 2	95 ± 5
RER	1.06 ± 0.1	1.12 ± 0.3
sBP, mmHg	181 ± 35	185 ± 34
dBP, mmHg	88 ± 14	79 ± 15
**Ventilatory threshold**
VO_2_, l/min	1.4 ± 0.3	1.5 ± 0.4
V_E_/VCO_2_	32 ± 3	29 ± 3*

VO_2_, oxygen consumption; HR, heart rate; V_E_, ventilation; SpO_2_, arterial oxygen saturation; RER, respiratory exchange ratio; sBP, systolic blood pressure; dBP, diastolic blood pressure; VCO_2_, carbon dioxide production; W, workload. ^*^P < 0.05, ^**^P < 0.01, ^***^P < 0.001: subjects with obesity vs. controls.

The univariate analysis showed that VO_2_peak was negatively correlated with V_E_/VCO_2_ at the VT (*R* = −0.51, *P* < 0.001), TAPSE at rest (*R* = −0.31, *P* < 0.05), α at rest (*R* = −0.29, *P* < 0.05) and the TAPSE/sPAP slope during exercise (*R* = −0.31, *P* < 0.05), but also positively correlated with total LM (*R* = 0.49, *P* < 0.01), leg LM (*R* = 0.51, *P* = 0.001), DL_NO_ (*R* = 0.44, *P* < 0.01) and DL_CO_ (*R* = 0.50, *P* < 0.001) or Dm (*R* = 0.44, *P* < 0.01) and Vc (*R* = 0.59, *P* < 0.001).

The multivariable analysis showed that only higher DL_NO_ (*P* < 0.01) and TAPSE/sPAP (*P* < 0.05) at rest, but also lower V_E_/VCO_2_ at VT (*P* < 0.01) were significantly associated with higher VO_2_peak (adjusted R^2^ of the model = 0.51, SEE = 7.5).

## Discussion

The present results suggest that obesity is associated with impaired RV-PA coupling as assessed by TAPSE/sPAP, and decreased pulmonary vascular reserve as assessed by steeper slope of mPAP-QI and decreased Vc. Correlation between exercise-induced decrease in TAPSE/sPAP and VO_2_peak as well as with the amount of body fat suggests that altered RV function adaptation to afterload in obesity may contribute to decreased exercise capacity.

In the present study, pulmonary vascular function was assessed by multipoint mPAP-Q plots. This approach allows for the detection of subtle changes in PVR that may be undetected by single resting measurements, and for the calculation of a derived resistive vessel distensibility factor α (14, 15). It is therefore reasonable to believe that pulmonary vascular function is altered in obesity, the more so that independently calculated Vc from lung diffusion capacity measurements was decreased as well. It is of interest that increased PAP at exercise was due to increased slope of mPAP/QI rather than to decreased resistive vessel distensibility factor α. This was previously observed in healthy subjects acutely exposed to low oxygen breathing (25) and in patients with early-stage hypertension (28) and may be explained by increased vessel tone due to an increase in medial thickness rather than by more extensive structural changes. Increased pulmonary vascular tone may be an early stage of more generalized pulmonary and systemic vasculopathy, possibly related to the release of pro-inflammatory cytokines (4, 29, 30). However, a reduction in lung volumes as typically reported in obesity (31) may also be invoked to explain higher PAP at any given level of flow without intrinsic changes in vascular distensibility.

The E/e' ratio was not different between obese subjects and controls during exercise. The absence of a significant exercise-induced increase in E/e' is in keeping with previous echocardiographic studies in healthy subjects (12, 13, 15). However, invasively measured pulmonary artery wedge pressure to estimate LAP has been reported in patients with obesity complaining of exercise dyspnea (9). Insufficient accuracy of LAP estimation from E/e' or insufficient maximal Q levels may account for the lack of a more sustained increase in LAP at exercise. However, this observation also may indicate that increased slope of mPAP-QI was not explained by altered diastolic function of the left ventricle.

Previous echocardiographic and magnetic resonance imaging studies have shown that obesity is associated with increased RV dimensions and hypertrophy, and depressed indices of systolic function (6–8). This was confirmed by an invasive study, which reported on a decreased RV/PA coupling assessed by gold standard Ees/Ea that was correlated to a decreased exercise capacity and decreased PA compliance (9). In the present study, RV-PA coupling was estimated by the TAPSE/sPAP ratio. This composite echocardiographic variable was initially proposed as an estimate of RV myocardial length-tension relationship, and as such showed to be of prognostic relevance in patients with heart failure (32, 33), pulmonary arterial hypertension (34) and chronic lung diseases (35). In these studies, the TAPSE/sPAP ratio was assumed to inform about RV-PA coupling, with TAPSE considered as a load-dependent surrogate of Ees and sPAP as an indirect estimate of Ea (33–35). The TAPSE/sPAP ratio has been shown to be superior to other composite echocardiographic indices in the assessment of RV-PA coupling and correlated to gold standard invasive (17) or indirectly assessed Ees/Ea ratios (33).

Age and fluid status have been shown to be significant drivers of the TAPSE/sPAP ratio (36). While the present results were probably little affected by aging, how much hypervolemia, typically observed in patients with obesity, affected the TAPSE/sPAP remains unknown in a context where RV function may adapt to preload changes. It is of interest that the TAPSE/sPAP ratio is reduced in mild systemic hypertension (37) like in the present study. Generalized vasculopathy and associated humoral factors (5, 29, 30) may affect the RV myocardium more than associated increase in afterload in obese patients. Decreased TAPSE/sPAP may be an indicator of obesity-induced cardiomyopathy (4).

The TAPSE/sPAP ratio was decreased in both obese subjects and controls during exercise. Exercise has been previously shown to decrease the TAPSE/sPAP ratio in healthy subjects (38). We therefore compared the TAPSE/sPAP ratio in obese subjects and controls at the maximum common value of QI. Higher QI at maximum exercise in controls was associated with a further decrease in TAPSE/sPAP ratio. The changes in TAPSE/sPAP ratio with exercise were correlated to the android fat mass which confirms that central obesity has a negative effect on RV function (8).

In the present study, DL_CO_, not DL_NO_ was decreased in obese subjects, but the difference with controls was smoothed out after correction for differences in V_A_ (21). This result is in keeping with previously reported unchanged or increased DL_CO_ in proportion to increased BMI in morbidly obese subjects (39). Derived Vc calculation which relies on non V_A_-corrected DL_CO_ and DL_NO_ was also decreased in obese subjects, supporting the notion that decreased pulmonary vascular reserve (increased slope of mPAP-Q) in obese subjects may be essentially determined by decreased lung volumes. This is further supported by the DL_NO_ and VE/VCO_2_ at VT emerging as independent predictors of VO_2_peak, as previously observed (12, 40).

Obese subjects presented with a significant reduction of body weight-corrected VO_2_peak, but still at the lower limit of predicted. There are studies reporting on a decreased VO_2_peak in relation to RV-PA uncoupling (shown be invasively measured Ees/Ea) (9) or a ventilatory limitation (shown by decreased V_E_/VCO_2_ and ventilatory reserve) (41). In the present study, V_E_/VCO_2_ was increased compared to controls in the subjects with obesity, but their VO_2_peak was not ventilatory-limited. However, TAPSE/sPAP was decreased at rest and at exercise and exercise-induced decrease in TAPSE/sPAP was independently correlated to VO_2_peak, suggesting that impairment of the RV-PA coupling in these patients may eventually limit exercise capacity. This is in line with previous studies showing that the RV-to-pulmonary circulation unit, including the capillary network but also the diffusion capacity and ventilatory efficiency are factors modulating the VO_2_peak in healthy subjects (11–13, 38, 40).

This study has limitations. First, the obese subject's sample was small and predominantly female. Sex may affect mPAP-Q relationships (15), RV structure and function (42), lung diffusion capacity (43) and VO_2_peak (44) but how this affects exercise capacity in obese subjects is not known. In the present study, although no sex differences were observed, the small study sample does not allow to draw definitive conclusions. Second, the study did not include arterial blood gases to better assess a ventilatory limitation to exercise by increased capnia (41). However, increased V_E_/VCO_2_ in our obese subjects makes this possibility unlikely. Third, RV-PA coupling was assessed by a non-invasive surrogate of RV Ees/Ea. However, recruitment of otherwise healthy obese subjects is difficult, particularly in our experience when an exercise test is involved. Therefore, adding a cardiac catheterization would have further limited recruitment. Fourth, sufficient quality echocardiography for the assessment of the RV and the pulmonary circulation is technically difficult, and this was the reason why more than half of obese candidates to our study could not be included. This may be a cause of bias, though with uncertainty how. Fifth, about half of the obese subjects had a history of hypertension, sleep apnea, prediabetes and hyperlipemia. We were unable to recruit more otherwise healthy obese subjects. In a *post hoc* evaluation, we did not find significant differences between otherwise healthy obese subjects and the others who presented with indices of metabolic syndrome and/or sleep apneas (all *p* > 0.05).

In conclusion, mild to moderate obesity is associated with an impairment of RV-PA coupling, which may affect exercise capacity. The clinical consequences of this finding deserve further investigation.

## Data availability statement

The raw data supporting the conclusions of this article will be made available by the authors, without undue reservation.

## Ethics statement

The studies involving human participants were reviewed and approved by the Local Ethical Committee of the University Hospital (Erasme EC Reference number: P2016/448; EC approval date: 2016-11-21). The patients/participants provided their written informed consent to participate in this study.

## Author contributions

NZ, VF, and RN contributed to conception and design of the study. NZ, YM, CS, KF, and MK contributed to the acquisition of data. NZ, KF, YM, VF, RN, CS, and MK contributed to analysis or interpretation of the data. NZ, KF, VF, and RN drafted the manuscript. MK, CS, and YM reviewed the article critically for intellectual content. All authors contributed to the article and approved the submitted version.

## Funding

YM was recipient of a fellowship of the *Fonds pour la Chirurgie cardiaque*, Belgium. SC was recipient of a Ph.D. grant of Innoviris Bridge, Brussels.

## Conflict of interest

The authors declare that the research was conducted in the absence of any commercial or financial relationships that could be construed as a potential conflict of interest.

## Publisher's note

All claims expressed in this article are solely those of the authors and do not necessarily represent those of their affiliated organizations, or those of the publisher, the editors and the reviewers. Any product that may be evaluated in this article, or claim that may be made by its manufacturer, is not guaranteed or endorsed by the publisher.
