# Corrigendum: Right ventricular-pulmonary arterial coupling impairment and exercise capacity in obese adults

**DOI:** 10.3389/fcvm.2022.1041285

**Published:** 2022-09-26

**Authors:** Zhou Na, Forton Kevin, Motoji Yoshiki, Scoubeau Corentin, Klass Malgorzata, Naeije Robert, Faoro Vitalie

**Affiliations:** ^1^Cardio-Pulmonary Exercise Laboratory, Faculty of Motor Science, Université Libre de Bruxelles, Brussels, Belgium; ^2^Department of Cardiology, Erasmus University Hospital, Brussels, Belgium; ^3^Laboratory of Applied Biology and Research Unit in Applied Neurophysiology, ULB Neuroscience Institute, Université Libre de Bruxelles, Brussels, Belgium; ^4^Laboratory for Biometry and Exercise Nutrition, Faculty of Motor Sciences, Université Libre de Bruxelles, Brussels, Belgium

**Keywords:** stress echocardiography, right ventricular-pulmonary arterial coupling, pulmonary circulation, pulmonary vascular resistance, pulmonary vascular reserve, VO_2_max = maximal oxygen uptake, obesity

In the published article, the author names were incorrectly written as Na Z, Kevin F, Yoshiki M, Corentin S, Malgorzata K, Robert N and Vitalie F. The correct author names are Zhou N, Forton K, Motoji Y, Schoubeau C, Klass M, Naeije R and Faoro V.

The authors apologize for this error and state that this does not change the scientific conclusions of the article in any way. The original article has been updated.

## Publisher's note

All claims expressed in this article are solely those of the authors and do not necessarily represent those of their affiliated organizations, or those of the publisher, the editors and the reviewers. Any product that may be evaluated in this article, or claim that may be made by its manufacturer, is not guaranteed or endorsed by the publisher.

